# “Power, control, strain”: Lay perceptions of health inequalities across England’s ‘North South divide’

**DOI:** 10.1016/j.socscimed.2024.117089

**Published:** 2024-07-08

**Authors:** Kate Bernard, Victoria J McGowan, Clare Bambra

**Affiliations:** Population Health Sciences Institute, Newcastle University, Ridley 1 Building, Newcastle-upon-Tyne, NE1 7RU, UK

## Abstract

People in the North of England live shorter, less healthy lives than those in the South. Despite the significance of this ‘North South health divide’, regional health inequalities in England are under-researched qualitatively. Existing literature on geographical inequalities in health is largely confined to the neighbourhood level, is quantitative, and consists of very little lay knowledge. The current study is the first to examine lay perspectives of health inequalities on a regional level: exploring how people living in two urban areas of the North and South of England experience and perceive the North South health divide – including its causes and solutions. Using three focus group discussions with a total of 34 participants, and conducting participatory analysis, we identified three key themes: ‘inequalities of power’, ‘lack of control over lived environment’ and ‘communities under strain’. Findings align with existing research on lay perspectives of health inequalities at the neighbourhood level – identifying a network of material-structural and psychosocial factors. Participants across both regions discussed political and economic structures as central to understanding regional health inequalities, supporting calls to adopt a political economy approach in understanding health and place. Deindustrialisation, unemployment, loss of community facilities, and disengagement from politics were more present in Northern narratives than Southern. Findings add important ‘social meaning’ to emerging research on the North South health divide, reinforcing the urgency of public health professionals’ recommendations for fair redistribution of power, wealth and resources to reduce regional health inequalities. In the context of government policy which diverges from public health evidence, this study sparks questions of how health inequalities research can intersect with wider social and political movements organising for systemic change.

## 1 Introduction

People in the North of England (North East, North West, and Yorkshire and Humber regions) live on average almost two years less than those in the rest of the country [Bambra, 2016]. Those in the North are 20% more likely to experience premature death - amounting to 1.5 million Northerners dying before their time since 1965 [Munford et al., 2023; Hacking et al. (2011). This ‘North South health divide’ in England is longstanding, documented both before and after the epidemiological transition [Bambra, 2016]. Similar regional health divides exist in countries like Italy, Brazil and the USA (Franzini and Giannoni, 2010; Szwarcwald et al., 2016) but England’s is particularly stark - now greater than the divide between the former West and East Germany [Bambra et al., 2014]. Evidence suggests the gap is worsening, especially among younger adults (Buchan et al., 2017; Kontopantelis et al., 2018; Munford et al., 2023).

Addressing this divide has become more urgent. Austerity measures introduced by the UK government in 2010 have disproportionately impacted Northern regions (Beatty and Fothergill, 2014; Pearce, 2013). The economic consequences of Brexit are expected to be regionally uneven (Los et al., 2017). Deaths from COVID-19 were 17% higher in Northern regions, as well as the North experiencing harsher lockdowns, hospital pressures, and greater mental health effects [Bambra et al., 2023]. The cost of living crisis is now hitting Northern regions hardest, severely impacting child poverty (Barnes et al., 2022; Rodrigues, 2023).

Recently there has been increased political interest in the North, evident in initiatives like the Northern Powerhouse (NPH, 2023), regional devolution deals (LGA, 2023) and the government’s ‘levelling up’ agenda – which aims to narrow the gap in Healthy Life Expectancy (HLE) by 2030 and increase HLE by five years by 2035 (GOV.UK, 2022, p. 200). However, critiques highlight limited action and funding of these developments (Camacho et al., 2023; Giovannini and Griggs, 2022; Tomaney, 2016).

Despite the significance of the North South divide, there has been no extensive academic study of its health facets [Bambra, 2016]. Few epidemiological studies describe England’s regional health inequality (Doran et al., 2004; Hacking et al., 2011; Kontopantelis et al., 2018; Möller et al., 2013), with a notable lack of qualitative research. Insights into the ‘social meaning’ of the divide are lacking, likely due to a focus on individual and localised factors in the study of geographical inequalities in health at the expense of a broader, more upstream lens [Bambra et al., 2019].

Avoiding the trap of silos, geographical health inequalities research must be contextualised (Collyer and Smith, 2020). Influential works by Dorling, Pickett, Whitehead and Marmot, along with critical epidemiology, have brought a structural perspective to the health inequalities research field more widely (Dahlgren and Whitehead, 2021; Dorling, 2013; Marmot and Bell, 2012; Wilkinson and Pickett, 2009). However, research focusing explicitly on the role of place has been slower in moving upstream. Traditionally, debates have centred on ‘context’ (economic, social, and physical environment) and ‘composition’ (demographic, behavioral, and socio-economic factors). Cummins et al. (2007) argue that the ‘false dualism’ between context and composition should be replaced by *relational approach* which recognises the reciprocal relationships between people and place. Building on this analysis, [Bambra et al., 2019] highlights that while context/composition research has advanced understanding of *local* neighbourhood effects on health, it has underemphasised broader factors acting at a regional, national and international scale – such as recessions, austerity, and health policy. This has minimised the crucial role of ‘macro’ political and economic structural drivers in our understanding of relationships between place and health. As [Bambra et al., 2019] argues, this reveals a need to ‘scale up’ our understanding of place and health towards a *political economy approach*.

Researchers increasingly recognise the importance of lay knowledge in understanding and tackling health inequalities. Over the past two decades, a growing number of studies have explored lay perspectives on health inequalities and the relationship between health and place, both in the UK and internationally [Garthwaite and Bambra, 2017; Garthwaite and Bambra, 2018; Airey (2003); Backett-Milburn et al. (2003); Bolam et al. (2004); Bridger et al. (2023); Davidson et al. (2006); Davidson et al. (2008); Fairbrother et al. (2022); Hansen et al. (2024); Hodgins et al. (2006); Kim et al. (2023); Macintyre et al. (2005); Mackenzie et al. (2017); Pinfold et al. (2024); Popay et al., 2003; Putland et al. (2011). This includes recent work focusing on lay perspectives of health inequalities’ solutions (Fergie et al., 2023; Smith et al., 2021; Subica and Brown, 2020). Despite methodological differences, there is a broad consensus that lay accounts integrate material-structural, psychosocial, and behavioral factors in explaining health inequalities. Upstream factors such as policy failures and misuse of power are consistently highlighted as key (Smith and Anderson, 2018). However, some studies find that people with greater lived experience of injustice often avoid applying structural understandings to their own situations to resist stigma and reassert individual agency (Bolam et al., 2004; Popay et al., 2003). Overall, lay perspectives closely mirror research-informed theories about health inequalities (Smith and Anderson, 2018). Importantly, studies so far focus only on individual or neighbourhood levels, with no exploration of regional, national, or global level perspectives.

This study attempts to close these gaps by exploring how people in two comparable urban areas in the North and South of England experience and perceive regional health inequalities, including causes and solutions. It aims to provide crucial ‘social meaning’ for understanding and tackling the North South health divide, and to expand our understanding of how health inequalities are perceived and experienced on a regional scale.

Resisting the ‘industrial’ nature of UK health inequalities research, criticised for its lack of political engagement, this study uses participatory methods in data collection and analysis to connect findings with levers for political change [Bambra et al., 2019]. The project itself sparked community action, demonstrating how qualitative health inequalities research can intersect with organising approaches to build power in communities. A deeper exploration of this process is beyond the scope of the current paper.

## 2 Methods

### 2.1 Setting

We identified field sites by comparing indices of deprivation across the 20% most deprived local authorities in England, focusing on areas in the North and South with similar patterns of deprivation but differing health outcomes. Manchester and the London Borough of Barking and Dagenham were chosen as both had similar patterns of deprivation, while health was worse in Manchester. The average life expectancy for women is 79.9 in Manchester, compared to 81.7 in Barking and Dagenham, and for men it is 75.5 versus 77.0. (Ministry of Housing, 2019). Within these areas we selected the more deprived council wards to understand the experiences of those most affected by inequalities in health, power and resources. Detailed context for the field sites is provided in Box 1 and online Appendix 1.

### 2.2 Study design and data collection

Qualitative focus group discussions were used to explore people’s perceptions and experiences of shared issues within the context of others’ views (Merriam, 2009). In 2022 a pilot focus group with ten participants in County Durham was conducted, followed by a preliminary thematic analysis and feedback gained from participants to improve data collection for 2023 [Bernard et al., 2023].

In June 2023, three focus group discussions were held in community centres in Manchester and Dagenham, facilitated by KB. Participants were recruited through visits to community centres and hubs including a gardening group, parent-toddler group, coffee mornings and ‘social supermarkets’. In Dagenham, one focus group with 12 participants was conducted in Heath. In Manchester, two focus groups were held, one in Beswick and one in Miles Platting (due to some participants’ limited travel ability), with a total of 22 participants. The focus groups were predominantly made up of women, with ages ranging from twenties to seventies. The Manchester focus groups were mainly made up of White British and Irish participants, while the Dagenham group was more diverse, including Black British, Black Caribbean, Black African, and Asian Pakistani participants. Three of the participants had prior experience of community work (one paid, two voluntary) and two had experience of political activism. In this paper, the focus groups are referred to as Focus Group North 1 (FGN1) in Beswick, Focus Group North 2 (FGN2) in Miles Platting, and Focus Group South 3 (FGS2) in Heath. Participants’ names have been changed for anonymity.

The focus group discussions were interactive workshops, beginning with a brief overview of the North-South health divide using short handouts outlining relevant statistics on differences in life expectancy and health outcomes. Participants then engaged in an introductory exercise where they drew their perceptions of North and South on a map of England. Key issues were collectively brainstormed and displayed on flipchart paper for reference during the session. Participants were asked their views on causes of health differences between the North and South, factors affecting health, and potential solutions for these health inequalities (see online Appendix 2 for topic guide). Discussions were transcribed verbatim and anonymised. Follow-up ‘analysis and action’ workshops were conducted after the focus group discussions.

Ethical approval was granted by XX University Faculty of Medical Sciences Ethics Committee on 19/04/2023 (Ref: 31367/2022).

### 2.3 Analysis

Analysis was informed by reflexive thematic analysis (Braun and Clarke, 2019), adapted to include participants’ active involvement through ‘analysis and action workshops’. During these workshops, participants collectively analysed transcripts from both North and South discussions and developed action steps from the research. Participants’ analysis strategies included making descriptive notes, listing recurring ideas, sorting them into categories and discussing and comparing responses within the group. The initial coding index was created using material from these workshops. MAXQDA software was used to code the transcripts (VERBISoftware, 2023), participants’ notes, memos, mind-maps, and personal reflections, using a predominantly inductive approach. Emerging patterns were discussed between KB and VJM after each analysis workshop. Final thematic coding was developed based on workshop results, KB’s reflections, review by VJM and CB, and sense checking with participants. This report underwent participant review, alongside a lay summary, and was edited according to their feedback.

### 2.4 Reflexivity

KB: I am a junior researcher from the North of England. My main area of reflection during this project was my positionality as a white middle-class researcher discussing health inequalities with people whose lives have been more affected by injustice than my own. This included a consciousness of the aforementioned ‘industrial’ nature of health inequalities research: prompting the steps taken to make the project participatory, and to support opportunity for tangible action in the community afterwards. Both before and during this project, I was working as a junior doctor in the NHS at a time of widespread industrial action and also organising in the health justice movement. These experiences have influenced my views of health inequalities as products of violent political and economic systems: which will have in turn shaped the lens through which I understood the data.

## 3 Findings

### 3.1 Inequalities of power

Both Northern and Southern groups expressed a profound sense of inequality between the public and those in political and economic power. Inequalities in power were deemed central to health both locally and nationally, with communities feeling ignored or harmed by political decisions. Those making these decisions were perceived as unaccountable to the public, and as prioritising profit over people’s health. Northern focus groups showed higher levels of political disengagement and consciousness of regional divides compared to Southern groups.

Firstly, those with the power to make decisions were perceived to be out of touch with local realities, particularly with regard to spending decisions. These decisions were often seen as disconnected from what would improve people’s lives, and instead understood as something for politicians to “glorify”. The ‘out of touch’ nature of decision makers was often tied to social divides, with a stronger geographical distance felt in Manchester: *“He [politician] lives in the posh part. He doesn’t know what is actually going on in the actual nitty-gritty parts of Manchester”* (Janet, FGN1). Speaking of decision-making in Westminster, Joanne (FGN1) expressed: *“The South don’t understand what the North are living in. It’s as simple as that.”*

Trust in politics was low, with many feeling disillusioned and excluded from meaningful dialogue. In Manchester’s analysis workshop, a key quote for the group was *“Beswick has a big voice, it just needs someone to listen”* (Sandra, FGN1). People felt disillusioned with the UK’s democracy, in which politicians were felt to be unaccountable to the general public – *“they’re above everybody”* (Shelena, FGS3). Some expressed disengagement from politics entirely, particularly evident in Manchester’s FGN1, where several in this group had not voted in recent local elections due to the introduction of photographic identity requirements and a sense of futility with the political system. This was a source of disagreement within FGN1 – with Mandy also stressing the importance of voter engagement: *“people didn’t come in [to vote]. That’s their own fault …. You don’t know, the person who did get in actually has been doing things around the area.”*

In Manchester’s FGN1, there was a pervasive feeling of long-term disappointment, with expressions like: *“we were promised all sorts weren’t we?”* (Josie, FGN1); *“if you live in a poor area you don’t count”* (Paula, FGN1). Participants also noted a sense of stigma attached to the area, with reactions like:*” When people say, like, “Where are you from?” If you say Beswick, you get the*, “Beswick?”“ (Josie, FGN1), indicating judgement. The experience of judgement, let-down, and neglect was seen as active marginalisation of the working class, particularly evident in Dagenham, with the government held responsible. In Shelena’s (FGS3) words, *“we are letting [the government] get away with murder”*. Participants discussed policies as deliberate choices leading to harm to health, as seen in Miriam’s statement about food poverty and the cost-of-living crisis:

“The government knows actually what they’re doing …. After this pandemic, it’s like, it is the government that is putting the rope and tying the knot on people’s neck, because they know exactly what they should do [to ease things], but they don’t want to do that … They continuously tie the rope to tighten it more and more.” (Miriam, FGS3).

Those in both North and South widely felt that those in power valued “making money” over the value they placed on people’s health. This was discussed in the context of both local and national policy decisions - including selling off green space, the ‘bedroom tax’, and privatisation of social housing - at the expense of health and wellbeing. Reacting to the closure of swimming pools and youth clubs, Miriam (FGS3) went on:

“the government don’t care about that, all they care about is to give private people the places to build their houses, their mansions and still increase so much money, you know?”

Indeed, wealth and political power were seen as going hand in hand. For Imran (FGS3), when considering cuts to funding of community facilities: *“Yes, it’s the priorities to the richest, who’s paying more money is getting the priority.”* In Manchester’s FGN1, as the group discussed inequalities in health both within and between the North and South, and where political power is held, Sandra stated simply *“Money talks. Tell me it doesn’t talk”*. These narratives go beyond discussing policy decisions, to identifying the political and economic dynamics underlying them.

Discussing solutions to inequalities in power, participants saw collective action as more powerful than acting individually. This came up in a range of examples – Imran (FGS3) and Alice (FGN2) discussed civil disobedience in resisting unwanted local developments, and Evelyn and Annetta (FGS3) used the example of a housing campaign that had gained numbers from publicity.

*“… if you look, when ITV did the homeless and the problems with the housing, and they were doing it all by themselves, and some of them had been doing it for 12, 15 years, as soon as it entered the media, things turned around for them. And there were more than 100 people.”* (Annetta, FGS3)

### 3.2 Lack of control over lived environment

Participants from both North and South described a lack of control over their lived environment – including their ability to breathe clean air, access outdoor spaces, and live in a decent home – with clear links to physical and mental health. These explanations were interwoven with the role of political and economic drivers in shaping their lived environment.

People in both Manchester and Dagenham described a decline of green space. This was discussed as due to “endless construction” (as coded in Manchester’s analysis workshop) - including new housing, office blocks, hotels, and car parks. Decline of green spaces was discussed alongside a sense of loss for the memories and connections these spaces held. In the rapidly gentrifying area of Miles Platting, participants described memories of socialising with friends and family in local green spaces, now replaced by private housing unaffordable to local people: *“I miss my park … All gone, all demolished”* (Tarek, FGN2). There was also a very physical sense of the space that people could freely access as shrinking or being encroached upon: *“There’s nowhere for us to go. We used to go and sit on the canal”* (Clare, FGN2).

People in both Manchester and Dagenham also described poor air quality as a result of traffic and building work, with frequent links to respiratory health:

*“You can’t walk up the street … Because of the dust. I can’t breathe. Then we’ve got a friend who lives right next door where they are driving in and out, in and out [building hotels]. They think she needs an oxygen tank. She’s a mess.”* (Erin, FGN2)

Crucially, people described these effects on their physical environment as something that felt beyond their control. New constructions were discussed as either being unaffordable to people in the area, or unwanted for their needs– an example of local decision making happening *to* rather than *with* the community, as discussed in Theme 1: *“They said they were going to build affordable housing in the borough. Affordable for who though?”* (Evelyn, FGS3).

As well as air quality and green space, people also expressed a lack of control with regard to their homes – including where they were able to live, and the conditions they were living in. Alice (FGN2) described a leak in her flat, and had been without heating or electricity for five weeks waiting for repairs:

“there’s a big company in this area … they just do whatever they want. They charge loads. They put the costs up all the time but they don’t do the basic maintenance. They then start going, “Oh dear. We’ve got an overspend this year.” You are like, “Yes, because you’ve just whipped all of it away in profit and now you are charging us even more to do basic things.”

The focus on profit being prioritised over people’s health resonates strongly in these statements, again with links to ‘inequalities of power’. The phrase “*they just do whatever they want*” encapsulates the sense of lack of control over lived environment. This lack of control also extended to *where* participants were able to live, in the context of rising rents and lack of social housing. In Dagenham many spoke of feeling trapped in social properties which were too small for their family:

*“Because the council were saying to me I’ve got to be in my property for, like, ten years before I get shifted. Now, I’ve done nine years and it was a hard nine years with my children. I’ve got four kids. Everyone keeps saying, “Go and see your MP” … I went to see [her] … she literally sided with the council … Told me, “Sorry, the council said there’s no homes, therefore there’s no homes.” … I literally went depressed, it led me to take tablets.”* (Shelena, FGS3)

In both North and South, privatisation of social housing was seen as directly responsible for its scarcity. Only in Manchester, however, was a regional divide discussed in reference to this process. “Rich Londoners” were specifically mentioned as the buyers who then let properties out to “make a mint”.

*“There’s not a lack of* housing. *It’s just that … the social houses that are around here are getting sold … They were council houses, three and four bedrooms and they are getting sold to single people that can afford to buy them from down South. They buy ours, rent them out to us and then take the money back down South”* (Joanne, FGN1)

Several participants experienced gentrification as a concerted effort to get the “normal people out”. The notion that “they” (those with wealth/political power) want “us” (the normal/working class people), out of the area, was recurrent in discussions – experienced as a systematic process of exclusion.

*“They are building new houses on the canal love … All private. It’s private. We are not allowed those. They want us out. They want the normal people out … Because Manchester, the City Centre is moving up. They want us lot out. [The government] are making too much money off these high-rises, so us lot, eventually, give us 10 years and there’ll be no estate. There will be all blocks*.” (Erin, FGN2)

Participants across the North and South felt meaningful participation in decision-making was central to regaining control over their environment and reducing regional health inequalities. Alice (FGN2) said “*I think we’d feel less powerless if there was a way of being like, “Hear our voices*.” Suggestions ranged from actions such as petitions and protests, to broader ideas of Citizens Assemblies and increased community ownership. Imran (FGS3) proposed defined roles for public involvement in government departments, and Edward (FGN1) mentioned community officers as a direct “*line of communication from all citizens to the council*” (already taking place in Miles Platting). People wanted their experiences to be heard, emphasizing face-to-face dialogue and public participation.

### 3.3 Communities under strain

Participants described their communities as being under severe economic and social strain, affecting community cohesion and health. Northern focus groups particularly felt a palpable sense of loss for the past alongside a sense of generalised decline.

Employment was a major source of strain. In Dagenham, main concerns centred on insecure work, zero-hour contracts, poor sickness arrangements, low wages, and the need for multiple jobs to get by - all impacting health. In Manchester, the primary issue was the lack of available of jobs. Dorothy (FGN1) contrasted this with her youth pre-1980s: “*you could walk into a job when I was young one week and walk out and get another job a week after”*. High unemployment was linked to deindustrialisation, with memories of factory, mine and mill closures and their effects on families. Edward (FGN1) recalled the area being “*derelict industrial land*” by the 1990s. Notably, there was a clear perception of deindustrialisation as a Northern experience. Joanne (FGN1) noted health divides as tied to historical class and wealth inequalities:

“Where down in London they weren’t quite working-class as us. The mines, the mills and things like that that we all had up here, they didn’t quite have them in London. They still had the posher kind of things compared to what we had here.”

High cost of living and inadequate wages were discussed as having knock-on effects for health - particularly stress, anxiety and depression. Imran (FGS3) expressed: “*Everyone is frightened, yes? Everything is going expensive, they are, like, pinning us down from every side*”.

This strain was discussed as impacting community cohesion and fuelling xenophobic narratives. In Manchester’s FGN1, with mainly White British/Irish participants, racial divisions were discussed most - particularly in relation to social housing. Dorothy (FGN1) felt pressured by the housing association to move to the outskirts of Manchester, fearing isolation from family and friends. She blamed immigration, describing “*too many people coming into the country*”, and the area being seen as a “*dumping centre*” by the government. For Janet (FGN1), *“[migrants] are being brought up into the poorer areas of the country … the government doesn’t think about the areas*.” However, other participants distanced themselves from these views. On deeper discussion, Sandra (FGN1) suggested that scarcity in social housing was “*creating racism*”, due to a sense of competition over resources. Kath and Josie described racism as a “*learned behaviour*” in the community, with attitudes passed down from parents to children, and fuelled by the media.

In the more diverse Dagenham focus group, which included participants with lived experience of migration, several participants also linked perceptions of resource distribution to racism. Annetta (FGS3) noted tension over migrants being perceived as taking limited resources: “*there is going to be tension because they’re seeing [migrants] as, “Oh, they’re coming in and … we’ve barely got enough.*” Annie (FGS3) saw such sentiments as being driven by fear: “*I travel by bus, and I’ve often stood at bus stops and listened to people talking, and they’re not being racist, they’re frightened, they’re frightened of things being taken away from them*.”

Community cohesion was discussed as being impacted by the decline of public spaces: summarised in Dagenham’s analysis workshop as “*no space to meet, talk*”. Mary from Manchester’s FGN1 highlighted the importance of these spaces for creating connections both between and within communities; giving an example of an event she ran as a community worker:

“Bringing everyone together … I did an event in a community centre and there were about 15 different cultures, and they all brought different food, and they all went away chatting to each other.”

The decline of public spaces was discussed as part of a broader reduction in community facilities including youth clubs, libraries, play groups, and swimming pools. Participants from across the North and South all put these trends down due to reduced funding. Despite this, several Dagenham participants praised local authority support. Imran (FGS3) noted increased community help “*We’ve got a lot of community and council help, and they are more active now*.” Annie (FGS3), who had previously lived in a Yorkshire town, contrasted this with her view of fewer facilities in Northern towns: “*Up north, I know there’s not as many [council] hubs and charities, and food banks as there is down here*.” Indeed, in Manchester (particularly FGN1), this perception of lack of amenities was confirmed, extending beyond community facilities to a sense of general decline: “*we’ve got nothing*” (Erin, FGN2). Dorothy (FGN1) and others described the exodus of services from their area (including shops, citizens advice bureau, job centre and credit union): “*Everything they’ve took away basically. Everything*.” The sense of generalised decline was more pronounced in Manchester than Dagenham.

Contrasting narratives highlighted solidarity, instead of division, amidst adversity and strain. In contrast to FGN1’s discussion on racial divisions, FGN2 participants viewed Manchester’s diversity as part of their strength in ‘sticking together’. Tarek (FGN2) called this social solidarity the “*old culture*”, having experienced this as stronger in Manchester than London. Many Manchester participants expressed genuine love for their area despite its challenges, valuing neighbourly support during tough times. Mandy (FGN1) recalled:

“I hope Josie doesn’t mind me saying, when you had the house fire the people that came out the back, “What do you need? What do you need?” You know, clothes. I thought, “I’m so glad I live here.”

In Manchester, social solidarity was seen as prevailing despite neglect from decision makers and inadequate community services. In Dagenham, mutual aid was less emphasised, possibly due to some participants feeling more able to access to community services (although this was not universal).

Declining health and social care services were also discussed as a strain for communities. Several described relationships with healthcare workers as more distant since the pandemic. Tarek (FGN2) remarked: “*Everything is going behind the walls. Even the GPs are behind the walls*.” Many reported difficulties accessing care, including GP appointments, A&E, and long delays for secondary care leading to worsening of health problems. Participants in both areas, some of whom were healthcare workers, attributed the health service’s difficulties to low wages for healthcare workers, understaffing, high demand, and long-term NHS underfunding. Privatisation and loss of staff after Brexit were also discussed. The health service was unanimously discussed in the context of decline, with the national government held responsible. Social care decline, especially impacting disabled people, was also highlighted. Gillian (FGS3) spoke with anger about the increasing difficulties in accessing both social care and welfare for her daughter with a severe learning disability. She linked these issues to government policies aimed at reducing disability benefits, framing this as a deliberate political choice:

“they made it absolutely clear that their intention with people with severe disabilities was to gradually whittle away the extra money they get, take it from them so that actually, at the end of the day, they will only have the same amount of money as anybody else who’s not able to work … that is their intention, that’s their stated intention.”

Participants felt that addressing regional health inequalities should be the government’s responsibility, not that of civil society. Edward (FGN1) stated: “*If you are asking who is responsible nationally [for improving regional health inequalities] it has to be I guess the government, not the charities*”. Policy suggestions included more preventative health measures, public sector pay rises, investment in social housing, regulation of private landlords, home insulation, and prioritising health inequalities across government departments. However, the national ‘levelling up’ agenda was viewed sceptically in both Manchester and Dagenham viewed as a politically expedient narrative, rather than a reality: “*It’s just another one of their slogans they are slinging in the air” (Erin, FGN2); “on the television, yes, yes … I don’t think it’s happening*” (Gillian, FGS3).

## 4 Discussion

### 4.1 Situating our findings within the ‘North South health divide’ literature

Our findings provide key qualitative insights into the North South health divide. Northern focus groups highlighted unemployment (as a legacy of deindustrialisation), decline of community services, political disengagement, and a strong sense of the North South divide. These narratives align with known greater impacts of austerity cuts in the North (Beatty and Fothergill, 2014; Pickett and Taylor-Robinson, 2021), trends of political disengagement (Quilter-Pinner et al., 2021) and the lasting effects of deindustrialisation for communities, identities, and experience of work (Linkon, 2018). Indeed, in Northern focus groups, there was a palpable sense of loss – of jobs, identity, community and stability. Narratives such as “we have nothing” and “everything has been taken away” echo Telford’s exploration of social and economic decline under neoliberalism in a North Eastern town (Telford, 2022).

Our findings align with existing academic perspectives on the North South health divide – including the Due North report, which describes the outcomes of an independent inquiry by a panel of public health practitioners [Whitehead et al., 2014]. In explaining the North South health divide, the Due North panel point to key differences in ‘power, poverty and resources’; ‘exposure to health damaging environments’ (e. g. unemployment); ‘chronic disease secondary to legacy of heavy industry and its decline’; and ‘opportunities to enjoy positive health factors’. Within the latter, the report highlights the notion of collective influence over resource use, strongly echoed by our participants. This connects to the significant literature on ‘collective control’ which shows that community control over decisions that affect their lives is a key determinant of health inequity (Popay et al., 2020; Whitehead et al., 2016).

The Due North report also aligns with our participants’ views on how to address regional health inequalities: including tackling economic inequality, increasing public influence over resource allocation, and strengthening the health sector’s role in promoting equity. In contrast, the current government’s Levelling Up agenda focuses mainly on lifestyle-based interventions to address regional health inequalities, despite acknowledging wider determinants like poor housing. This reflects ‘lifestyle drift’ in policy – where policies and interventions consistently focus on ‘bad behaviours’, even when acknowledging wider determinants causing health inequalities (Popay et al., 2010). The Levelling Up agenda aims to close the healthy life expectancy gap by addressing diet, smoking, alcohol and drugs, and diagnostic backlogs, with interventions like an app rewarding users for increasing their step count (GOV.UK, 2022). This displays contrast with what both public and professionals believe is needed to address regional health inequalities. Indeed, there are concerns that ‘levelling up’ might widen inequalities further - due to uneven funding distribution between North and South (Camacho et al., 2023), top-down disregard for local democratic links (Giovannini and Griggs, 2022), and failure to deliver schemes on the ground (Williams et al., 2024).

### 4.2 Situating our findings within literature on lay perspectives of health inequalities

Our findings provide insight into lay perspectives of health inequalities at the regional level. Our themes share much similarity with previous neighbourhood-level studies – revealing multi-layered narratives of material-structural conditions and psychosocial factors.

Psychosocial pathways, explaining how structural conditions impact health, were consistent with lay accounts at the neighbourhood level. Smith and Anderson (2018) identified factors such as stigma, strain, declining social networks and political disengagement, evident in our themes. Participants highlighted housing and poverty as major factors affecting mental health, echoing previous studies (Davidson et al., 2006, 2008; Parry et al., 2007). Moreover, discussions of place-based stigma align with findings from [Garthwaite and Bambra, 2018], Popay et al. (2003) and Airey (2003) among others.

Our findings broadly echo narratives around material-structural conditions found in previous studies on lay perspectives, whilst displaying slightly more emphasis on ‘upstream’ factors than some neighbourhood-level accounts (Davidson et al., 2008; Garnham, 2015; Mackenzie et al., 2017). Factors such as unemployment, poor housing, community neglect, poverty were all discussed as in previous studies - including a sense that those in positions of power had deliberately neglected certain areas, to the benefit of others (Smith and Anderson, 2018). In our study, many participants also went beyond naming these factors, to articulating the role of policy drivers such as deindustrialisation, welfare state retrenchment, and public sector cuts in producing and sustaining these conditions - evident across all themes. In Theme 1, Mariam discusses the cuts to community facilities as a political choice, taking place as the wealth gap widens. In Theme 2, Erin and Joanne link the health effects of lack of social housing to broader trends of privatisation. In Theme 3, Gillian describes how governments “stated intention” to reduce disability benefits impacted her daughter’s care. Indeed, not only do participants link these experiences to policy decisions, but begin to frame these decisions as shaped by political and economic structures (where “money talks”, and wealth is closely tied to political power), and influenced by those who stand to gain from inequalities continuing (through extraction of profit). This is summed up in Erin’s (FGN2) narratives around gentrification as a symptom of a profit-driven political system: “*They want us lot out. [The government] are making too much money off these high-rises.”* This higher level of structural discourse shows parallels with only a few previous studies at the neighbourhood level, notably Mackenzie et al. (2017) and Garnham (2015).

Mackenzie et al.’s (2017) study in West Central Scotland and Garnham’s (2015) study in a Scottish town both heavily emphasise the role of politics, deindustrialisation, and economic policy in explaining ill health. In Garnham’s study, participants discussed upstream factors around distribution of power and resources particularly when referencing regional divides: expressing frustration with government neglect of regions outside of the South of England.

*“They couldn’t care less, north, as was said, of the Watford Gap. They’re not even interested in the North of England or anywhere, they’re only interested in a little bit down there, they’re not interested in anywhere else … They’re the biggest thieves out! … So there’s something sadly wrong with government, isn’t there?”* (Garnham, 2015, p. 328)

Lay explanations for health inequalities are influenced by framing. Mackenzie et al. (2017) suggest that ‘foregrounding’ of macro-level factors like austerity in their study enabled their participants to voice more upstream understandings of ill-health. It also may be that framing discussions about health inequalities regionally, rather than locally, encourages upstream narratives - as seen with our participants. This is difficult to ascertain however - given that findings on lay perspectives of health inequalities also vary significantly with methodology. Davidson et al. (2006) consider the role of focus groups in contributing to structural narratives within their study – arguing focus group discussions can facilitate more ‘collective accounts’; transforming ‘personal troubles’ into ‘public issues’ (Davidson et al., 2006, p. 2180). It may be that focus group discussions had a similar effect in our study. Using other methodologies like ethnography could provide further insights into lay perspectives of regional health inequalities.

Additionally, the *language* used by our participants aligns with existing literature on lay perspectives at the neighbourhood level. For example, in Mackenzie et al.’s (2017) study:

“almost all participants told of austerity and labour markets shaped to benefit the affluent. ‘Neoliberal political attack’ was rendered almost viscerally in some accounts; Marion said, “I think they’re trying to kill folk aff.” (Mackenzie et al., 2017, p. 237).

In a similar way, many participants in our study used language of violence to describe the impact of political and economic structures on health, such as: “[the government] are getting away with murder”; “tying the knot on people’s neck”. That such language forms a key part of lay understandings, particularly in studies conducted during or after austerity, is important to heed. It aligns with calls for wider use of the concept of ‘socio-structural violence’ to understand health inequalities more fully. Jones and McCormack call for moving away from depoliticised language and using the language of structural violence when describing and analysing health inequalities – as a way to better reflect the experiences of those affected, and to be more effective in addressing the issues [Jones and McCormack, 2015]. Raphael et al. (2022) suggest that employing ‘polemic, anger-mobilising’ language like ‘social murder’ and ‘structural violence’ can better mobilise the public to demand government action. The fact that our participants already use such language highlights the importance for health inequalities researchers to carefully consider the framing of their discourse.

### 4.3 Situating our findings within the literature on how health and place interrelate

Our findings support the call to adopt a *political economy approach* to analyse health and place as suggested by [Bambra et al., 2019]. Lay participants in both regions emphasised macro-level political and economic drivers when discussing the relationship between health and place, rather than solely focusing on contextual or compositional factors. They often discussed contextual factors as closely intertwined with wider structural factors. There is alignment between calls by academics to ‘scale up’ our analyses of how place and health interrelate, and the perspectives of our lay participants - whose analyses of health inequalities at the regional level already appear considerably ‘scaled up’ in nature.

### 4.4 Implications

Our findings provide important social meaning to emerging research on the North South health divide, reinforcing the urgency of public health professionals’ recommendations for fair redistribution of power, wealth and resources to reduce health inequalities in England. They also add to the established consensus among academics and the public that political and economic structures are fundamental to producing and sustaining health inequalities. However, current government policies to address regional health inequalities contradict what both the public and professionals believe is necessary. Instead, they exhibit ‘lifestyle drift’, reflecting a broader disconnect between public health evidence and policy decision-making (Popay et al., 2010).

In the context of this status quo, our findings emphasise the need for the health inequalities research community to reframe our questions and re-strategise our action. Suggestions to reframe questions have included analysing policies and processes that create injustice and focusing on solutions (Smith and Anderson, 2018). Recently, this has extended to new frameworks that explicitly identify capitalism and the dynamics of capital accumulation as the “particular political and economic systems that are at stake” when we discuss health inequalities (Freudenberg, 2021; Raphael and Bryant, 2023; Singh and Hickel, 2023). Proposals for re-strategising action include adopting participatory practices for community empowerment and expanding public health advocacy (Smith and Anderson, 2018; Smith et al., 2015). Acknowledging the limitations of traditional public health advocacy (broadly defined as work to influence policy makers on behalf of communities), Raphael et al. argue for moving beyond these strategies, towards “mobilising the public to force government action” (Raphael et al., 2022, p. 135). As such, Freudenberg (2021), Raphael et al. (2022), and Singh and Hickel (2023) challenge us to think more expansively about how the health inequalities research community can ally with, and actively support, social and political movements fighting to address the intersectional root causes of health inequalities. Singh and Hickel (2023) put forward the key role of healthcare researchers and practitioners in supporting ‘organised political struggle’ towards solutions to health inequalities.

Indeed, our findings underline the growing need for communities, across both the North and South of England, to ‘get organised’. Unlike advocacy, organising focuses on building power at the community level. As McAlevey articulates (2016), the theory of power behind organising is mass, inclusive and collective, involving ordinary people rather than elites to achieve wins. It begins by building solidarity among people experiencing interconnected oppressions, and deepening people’s understandings of the power structures creating and sustaining those oppressions. We witnessed this process during our study, leading to collective action in the communities following the research: with participants taking steps to form a tenants union branch in Dagenham, and a new community group in Manchester. Alongside advocacy, there is an argument that health inequalities researchers (especially those employing qualitative methods) ought to skill up on organising, and allow organising approaches to shape our research and action (Sinnott et al., 2023). Increased use of participatory action research methods as advocated by our colleagues could facilitate the integration of organising approaches into health inequalities research.

Addressing how health inequalities academics can better ally with political movements merits a separate paper. However, at minimum efforts should focus on enhancing connections between the health inequalities research community and political organisations and movements with shared goals. This could entail broadening political education among health inequalities researchers and strengthening ties with grassroots campaigners and organisers taking action for social justice, both locally and nationally. In the longer term, it could involve conducting research to more directly resource and support campaigns addressing health injustice and collaborating in coalition with groups organising for progressive systemic change.

### 4.5 Limitations

Our study had several limitations. Firstly, the Manchester sample were mainly White British or Irish, failing to adequately represent the area’s demographics. Despite efforts to address this through recruitment surveys and outreach in diverse areas, success was limited. This may have been due to our focus on community centres as a site for recruitment, which in Beswick seem to have been frequented more by White British individuals. Consequently, important perspectives of individuals from other ethnic backgrounds in Beswick will have been overlooked, impacting our discussions; including those on community cohesion.

Secondly, although recommended by Smith and Anderson (2018) to include people from affluent areas, time and resource constraints prevented us from conducting additional focus groups with this demographic. However, we followed their suggestion to employ participatory practices, and also avoided binary, and potentially stigmatising, representations of the ‘North as unhealthy’, and the ‘South as healthy’. Instead, we emphasised nuanced health experiences within communities and explored how people engage with underlying factors driving health inequalities. Rather than focusing solely on causes, we also discussed solutions at both policy and community levels.

## 5 Conclusion

This study examined lay experiences and perceptions of England’s North South health divide through focus group discussions in two urban areas of both regions, identifying three themes: ‘inequalities in power’, ‘lack of control over lived environment’, and ‘communities under strain’. Findings align with existing research on lay perspectives of health inequalities at the neighbourhood level, revealing multi-layered narratives of material-structural conditions and psychosocial factors. Participants across both regions highlighted political and economic structures as central to understanding regional health inequalities, supporting calls to adopt a political economy approach in understanding health and place. The study underscores the urgency of fair redistribution of power, wealth and resources to address England’s health inequalities, especially in light of government policies diverging from public health evidence. It prompts consideration of how health inequalities research can align with organising approaches for political change. We echo the calls of our colleagues, that if we are to work in solidarity with our participants, and help them build power in communities, then action-oriented research in health inequalities needs to become the norm not the exception.

## Supplementary Material

**Appendix A. Supplementary data**

Supplementary data to this article can be found online at https://doi.org/10.1016/j.socscimed.2024.117089.

## Data Availability

The authors do not have permission to share data.

## Box 1 Field site context

*Manchester (FGN1):* Beswick is an inner-city area of Manchester. It is in the top 20 most deprived council wards in Manchester according to indices of multiple deprivation (IMD) (GOV.UK, 2019).

*Manchester (FGN2)*: Miles Platting is a rapidly gentrifying inner-city area of Manchester. It is also within the top 20 most deprived council wards in Manchester according to IMD, with an IMD score higher than that of Beswick.

*Dagenham (FGS3)*: Heath and its surrounding wards are in the London Borough of Barking and Dagenham, around 9 miles East of Central London. Barking and Dagenham has the highest IMD score in London, and Heath is in the most deprived half of wards in the borough.
